# Identifying interactions in the time and frequency domains in local and global networks - A Granger Causality Approach

**DOI:** 10.1186/1471-2105-11-337

**Published:** 2010-06-21

**Authors:** Cunlu Zou, Christophe Ladroue, Shuixia Guo, Jianfeng Feng

**Affiliations:** 1 Department of Computer Science, University of Warwick, Coventry, UK; 2 Department of Mathematics, Hunan Normal University, Changsha, China; 3 Centre for Computational Systems Biology, Fudan University, Shanghai, China

## Abstract

**Background:**

Reverse-engineering approaches such as Bayesian network inference, ordinary differential equations (ODEs) and information theory are widely applied to deriving causal relationships among different elements such as genes, proteins, metabolites, neurons, brain areas and so on, based upon multi-dimensional spatial and temporal data. There are several well-established reverse-engineering approaches to explore causal relationships in a dynamic network, such as ordinary differential equations (ODE), Bayesian networks, information theory and Granger Causality.

**Results:**

Here we focused on Granger causality both in the time and frequency domain and in local and global networks, and applied our approach to experimental data (genes and proteins). For a small gene network, Granger causality outperformed all the other three approaches mentioned above. A global protein network of 812 proteins was reconstructed, using a novel approach. The obtained results fitted well with known experimental findings and predicted many experimentally testable results. In addition to interactions in the time domain, interactions in the frequency domain were also recovered.

**Conclusions:**

The results on the proteomic data and gene data confirm that Granger causality is a simple and accurate approach to recover the network structure. Our approach is general and can be easily applied to other types of temporal data.

## Background

One of the most fundamental issues in computational biology is to reliably and accurately uncover the network structure of elements (genes, proteins, metabolites, neurons and brain areas etc.), based upon high throughput data [1,2]. There are several well-established reverse-engineering approaches to explore causal relationships in a dynamic network, such as ordinary differential equations (ODE), Bayesian networks, information theory and Granger Causality.

The notion of Granger causality, which was first introduced by Wiener and Granger [3-5], proposed that there is a causal influence from one time series to another if the prediction of one time series is improved with the knowledge of the second one. The prediction is made in terms of an auto-regressive model. In addition, Granger causality has the advantage of having a corresponding frequency domain decomposition so that one can clearly find at which frequencies two elements interact with each other. Granger's conception of causality has been widely and successfully applied in the econometrics literature and recently in the biological literature [6-11].

Considering the four different approaches to the same problem, a natural question is to investigate which should be preferred. In a previous paper [12], we presented a comparison study of Granger causality and dynamic Bayesian network inference approaches. The result showed that Granger causality outperformed the dynamic Bayesian network inference when the time series were long enough because the Granger causality was then able to detect weak interactions. In a recent *Cell *paper [13,14], the authors carried out a systematic comparison between the ODE, Bayesian and information theoretic approaches for a small synthesized gene network in the yeast (Saccharomyces cerevisiae). The authors concluded that the ODE was the best approaches amongst those three. We have applied our conventional Granger causality approach on the same recorded time-series and found that the results derived by it were better than all the other three approaches' in the original paper. A small network of seven previously investigated proteins [15] was also re-constructed. Interestingly, the two important proteins DDX5 and RFC1 found in experiments were at the top of the re-constructed network. Frequency domain results were analyzed and they indicated that DDX5 and BAG2 interacted at a frequency of one cycle per three hours.

Due to the complexity of biological processes, in order to capture the dynamics of complex systems and investigate the functions of genes and neurons in detail, it is much better to treat the network as a whole instead of analyzing a very limited portion of it. Until now, most of the analysis tools currently used for the whole network are based on clustering algorithms. These algorithms attempt to locate groups of genes that have similar expression patterns over a set of experiments. Such analysis has proven to be useful in discovering genes that are co-regulated and/or have similar function. A more ambitious goal for analysis is revealing the structure of the transcriptional regulation process, for example, for a given transcription factor, could we find all its upstream and downstream transcription factors? This is clearly a challenging and fascinating problem.

Most popular approaches, such as Granger causality, are powerful in cases where the length of the time series is much larger than the number of variables, which is exactly the reverse of the situation commonly found in microarray experiments, for which relatively short time series are measured over tens of thousands of genes or proteins. The real difficulty comes from the fact that when the dimension is larger than the length of time series, the design matrix of predictors is rectangular, having more columns than rows; in such case, the model is under-determined and cannot be uniquely fitted. Bayesian network is a graph-based model of joint multivariate probability distributions that captures properties of conditional independence between variables, but as it requires a large number of parameters and assumptions upon the variable distribution, it also quickly becomes intractable for large networks. Keeping these limitations in mind, it is still an important task to developing methodologies that are both statistically sound and computationally tractable to make a full use of the wealth of data now at our disposal. In order to tackle this problem, we propose a new framework: Global Granger Causality (GGC) This framework builds on the use of partial Granger causality which was illustrated in our previous paper [16]. We first construct an initial sparse network by considering all possible links by computing bivariate pair-wise Granger causality. Once we identify such a network structure, there is uncertainty about the true causal structure; we need to check whether the links appearing in pairwise causality are direct or indirect. We do so by computing GGC step by step. If a link is found to be an indirect relationship in the sense of GGC, we delete such a link from the initial network. Theoretically, iterating the procedure will remove all indirect links and only direct connections will remain. The advantage of such an approach is obvious. By explicitly taking more sources into account, it provides a less biased structure of the network due to latent variables than in a small network as described above. It also provides information on the ancestors and descendents of key elements such as DDX and RFC1 in our network. The results can then guide experimentalists to investigate the properties of a small subset of specific proteins.

The rest of the article is divided in two sections. First, in the method sections, we introduce Granger causality in details, as well as its formulation in the frequency domain. We also describe global Granger causality, the new procedure for applying Granger causality to large networks. Next, in the result section, we apply our method on small (local) and large (global) networks. In both cases, simulations and actual biological data (gene and protein time-series) are used and results discussed. And we also provide a theoretical proof of its reliability

## Method

A measurement of Causal influence for time series was first proposed by Wiener-Granger. We define the causal influence of one time series on another by quantifying the improvement made on the prediction of a time series when we incorporate the knowledge of a second one. Granger implemented this notion in the context of linear vector auto-regression (VAR) model of stochastic processes. In the AR model, the variance of the prediction error is used to test the prediction improvement. For instance, consider two time series; if the variance of the autoregressive prediction error of the first time series at the present time is reduced by the inclusion of past measurements from the second time series, then one can conclude that the second time series have a causal influence on the first one. Geweke [17,18] decomposed the VAR process into the frequency domain and converted the causality measurement into a spectral representation which made the interpretation more appealing.

The pair-wise analysis introduced above can only be applied to bivariate time series. For more than two time series, a time series can have a direct or indirect causal influence to other time series. In this case, pairwise analysis is misleading and not sufficient to reveal whether the causal interaction between a pair is direct or indirect. In order to distinguish direct and indirect causal effects, one introduces conditional Granger causality which takes account of the other time series effect. In the following we present an analysis on how to define the conditional Granger causality on an ARIMA (autoregressive integrated moving average) model. ARIMA is a generalization of an ARMA model. The model is generally referred to as an ARIMA(p,d,q) model where p, d, and q are integers greater than or equal to zero and refer to the order of the model. Given a time series of data X_*t*_, an ARIMA(p,d,q) model is given by:(1)

Where L is the lag operator, the error term *ε*_*t *_has normal distribution with 0 mean.

### Conditional Granger Causality in the time domain

Giving two time series **X**_*t *_and **Z**_*t *_and their k^th ^and m^th ^order differences Δ^*k*^**X**_*t *_and Δ^*m*^**Z**_*t *_(without loss of generality, we assume that m = k from now on), the joint autoregressive representation for Δ^*k*^**X**_*t *_and Δ^*k*^**Z**_*t *_by using the knowledge of their past measurement can be expressed as(2)

The noise covariance matrix for the system can be represented as(3)

where var and cov represent variance and co-variance respectively. Incorporating the knowledge of the third time series, the vector autoregressive mode involving the three time series Δ^*k*^**X**_*t*_, Δ^*k*^**Y**_*t *_and Δ^*k*^**Z**_*t *_can be represented as(4)

And the noise covariance matrix for the above system is(5)

where **ε**_*it*_, *i *= 1,2,⋯,5 are the prediction errors, which are uncorrelated over time. If we rewrite equation (2) and equation (4) in terms of X, Y and Z themselves, we see that whether a coefficient vanishes or not is almost unchanged. Hence it is safe to say that the conditional Granger causality form **Y **to **X **conditional on **Z **can be defined as (see [19] for the classical definition)(6)

When the causal influence from **Y **to **X **is entirely mediated by **Z**, the coefficient *b*_2*i *_is uniformly zero, and the two auto-regressive models for two or three time series will be exactly same, thus we can get var(ε_1*t*_) = var(ε_3*t*_). We then have *F*_Y→X|Z _= 0, which means **Y **can not further improve the prediction of **X **including past measurements of **Y **conditional on **Z**. In other words, **Y **doesn't have an influence on **X**. For var(**ε**_1*t*_) > var(**ε**_3*t*_), *F*_Y→X|Z _> 0 and therefore there is a direct influence from **Y **to **X**, conditional on the past measurements of **Z**.

### Conditional Granger Causality in the frequency domain

To derive the spectral decomposition of the time domain conditional Granger causality, we multiply the normalization matrix(7)

to both side of equation (2) and rewrite it in terms of the lag operator L. *I *is identity matrix. The normalized equations are represented as:(8)

Then we can apply the same normalization procedure to the equation (4) multiplying the matrix(9)

where(10)

and(11)

to both sides of equation (4) and rewrite it in terms of the lag operator(12)

After Fourier transforming equation (8) and (12), we can rewrite them in the following representations(13)(14)

where Δ(λ, k) is the Fourier transform of the difference operator Δ^k^. Therefore, for ARIMA and ARMA model in the frequency domain, their causality is identical. This is in agreement with our conclusions in the time domain causality and in general the Kolmogorov identity holds true, that is: integrating the frequency-domain Granger causality over all frequencies yields the time-domain Granger causality.

### Global Granger Causality

Partial Granger causality (PGC) provides an accurate description of the internal dynamics of the system when the number of nodes is much smaller than the length of recorded time series. However, when the number of nodes increases, especially when they are larger than the length of time series, a 'curse of dimension' immediately arises, it is a situation for which usual methods break down.

**Figure 1:**
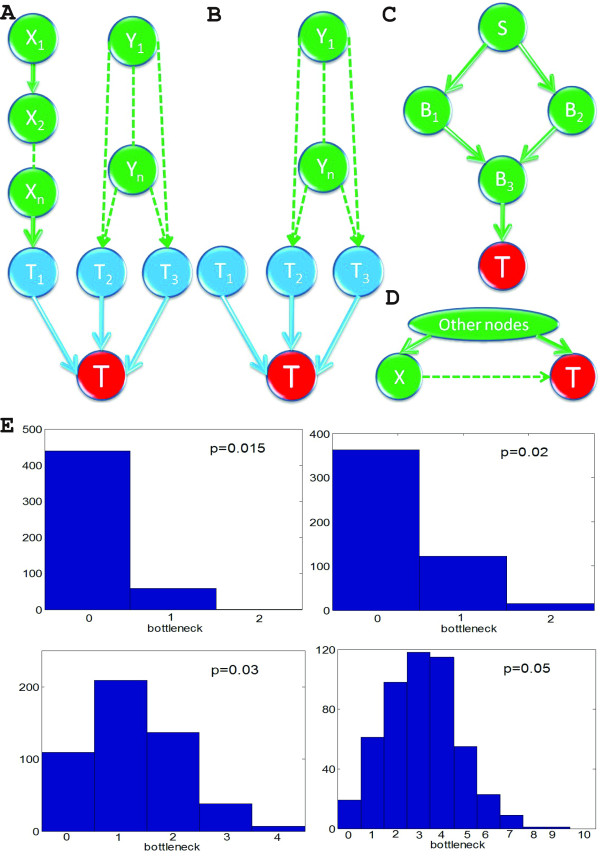
**Global Granger causality approach**. (**A**) Ancestors of target node T, A_0_(T) = {T_1_, T_2_, T_3_, X_1_,...,X_n_, Y_1_,...,Y_n_}. T_1_, T_2_, T_3 _are direct ancestors to target T. {X_1_,...,X_n_} connect to T through a single pathway, thus, {X_1_,...,X_n_} are not direct ancestors to target T. {Y_1_,...,Y_n_} connect to T through two distinctive pathways (**B**) {X_1_,...,X_n_} can be removed by Granger-conditioning on a single node, A_1_(T) = { T_1_,T_2_,T_3_,Y_1_,...,Y_n_}. (**C**) S is connected to T through two different paths, both {B_1_,B_2_} and {B_3_} are sections from S to T, but {B_3_} is the bottleneck. (**D**) There may exist other common drives to the observed nodes X and T, we assume the partial Granger causality can delete the influence of such drive and exclude such case in our analysis. (**E**) Histograms of the number of bottleneck for a variety of connection probability p for N = 100 and 500 simulations.

Here we propose the following Global Granger Causality (GGC) algorithm to tackle this problem. The general idea is as follows: if we could find all ancestors of a given target T, the whole network could be reconstructed. Hence for a given target T, we want to find all directed ancestors (parents of target T). For illustration, a small subset of the whole network, which contains target T and all its ancestors, is shown in the Fig. 1A. We assume that each nodes from {X_1_,...X_n_} has only a single pathway to target T, and each nodes from {Y_1_,...,Y_n_} has two distinct pathways to target T. From Fig. 1A, we can find the parents of target T are T_1_, T_2_, T_3_.

The detailed algorithm is illustrated as follows:

First, apply the bivariate pair-wise Granger causality to find all of the ancestors of the target T. This set is denoted A_0_(T). In theory, we can detect all possible Granger-causal links in this procedure, both direct and indirect. In Fig. 1A, A_0_(T) = {T_1_, T_2_, T_3_, X_1_,...X_n_, Y_1_,...,Y_n_}.

Secondly, we identify whether the links detected in step 1 are direct or indirect. For such a purpose, we carry out the following iterative procedures.

(I) For each node in A_0_(T), compute the partial Granger causalities conditioned on all other single nodes in the A_0_(T). If the relationship vanishes, delete this node from the initial network and obtain the 1-stage network. After this procedure, all indirect links conditioned on one single node have been removed. In Fig. 1A, {X_1_,...X_n_} are deleted from A_0_(T), denoting the remaining set as A_1_(T) ={T_1_, T_2_, T_3_, Y_1_,...,Y_n_}. This is proved in **Lemma 1 **of Discussion section.

(II) For each node in A_1_(T), compute the partial Granger causalities conditioned on all possible pairs in A_1_(T). We obtain the 2-stage network in where all indirect links conditioned on a pair of nodes have been removed. In Fig. 1B, {Y_1_,...,Y_n_} is further deleted from A_1_(T), denoting the remaining set as A_2_(T) ={T_1_, T_2_, T_3_}.

(III) Continue the procedure above until we can not remove any nodes from A_k_(T).

The rationale is as follows: if the usual Granger causality from Y → X is large but significantly decreases when conditioned on a third signal Z (F_Y→X|Z _), then the connection Y→X is only indirect and should be discarded. We use this principle to find the direct ancestors (signals acting on a target X with no intermediate) of each nodes. At step 0, we search for all signals Y such that F_Y→X _is large. We call A_0 _this collection of candidate ancestors. At step 1, we filter this set further with keeping the signals Y∈A_0 _such that F_Y→X|Z _is still large for all Z∈A_0_. We call A_1 _this new set and carry on the procedure by conditioning on groups of 2, then 3 etc. signals from the previous set until such an operation is not possible (the size of A_i _decreases or stabilizes at each iteration). The result is a list of direct ancestors for each node, which we aggregate to produce the global network.

## Result and Discussions

### Local Network: Synthesized Data

To illustrate the conditional Granger causality approach in both time and frequency domains, a simple multivariate model with fixed coefficients which has been discussed in previous ([9,16]) papers is tested first.

Suppose we have 5 simultaneously recorded time series generated according to the equations:(15)

**Figure 2:**
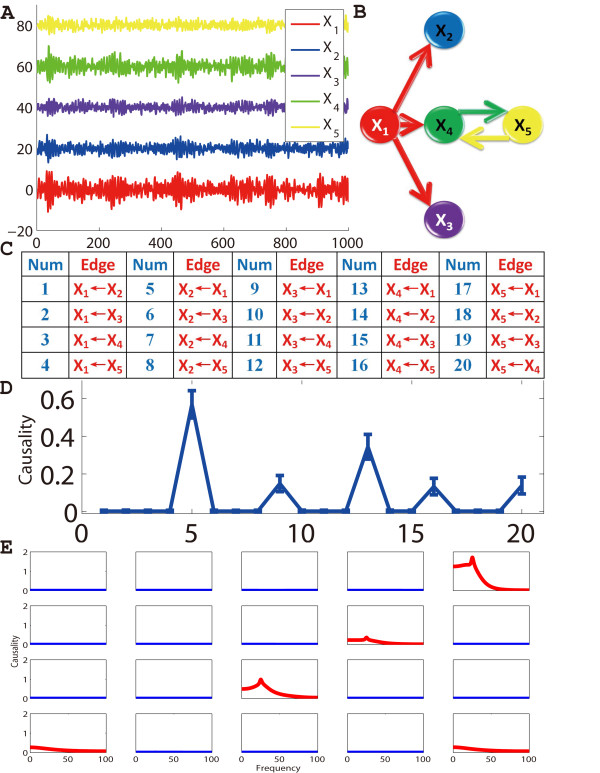
**Conditional Granger causality approach applied on a simple linear toy model**. (**A**) Five time series are simultaneously generated, and the length of each time series is 1000. *X*_2_, *X*_3_, *X*_4 _and *X*_5 _are shifted upward for visualization purpose. (**B**) For visualization purpose, all directed edges (causalities) are sorted and enumerated into the table. (**C**) The derived network structure by using conditional Granger causality approach. (**D**) The 95% confidence intervals graph for all the possible directed connections derived by conditional Granger causality. (**E**) Granger causality results in frequency domain.

Where *n *is the time, and [*ε*_1_, *ε*_2_, *ε*_3_, *ε*_4_, *ε*_5_] are independent Gaussian white noise processes with zero means and unit variances. From the equations, we see that *X*_1_(*n*) is a cause of *X*_2_(*n*), *X*_3_(*n*) and *X*_4_(*n*), and *X*_4_(*n*) and *X*_5_(*n*) share a feedback loop with each other. Fig. 2A shows an example of the time trace of 5 time series. We obtain 95% confidence intervals by bootstrapping: we simulated the fitted vector autoregressive model to generate a data set of 1000 realizations of 1000 time points with a sampling rate 200 Hz and used their statistics for estimating the confidence intervals [20]. (Fig. 2D). An ARMA (Auto-Regressive Moving Average) model was used to fit the data, and samples were drawn from this fitted ARMA model. To depict all causal relationships in a single figure, we enumerated them in a table as shown in Fig. 2C. According to the confidence intervals, one can derive the network's structure as shown in Fig. 2B. From the result, the Granger causality approach correctly recovered the pattern of the connectivity in this toy model. Furthermore, we applied the conditional Granger causality approach on frequency domain as shown in Fig. 2E. The causal relationships from *X*_1 _to *X*_2_, *X*_3 _and *X*_4 _show strong interactions at around 25 Hz.

### Local Network: A yeast synthetic network of five genes

A recent *Cell *paper by Irene Cantone *et al*. [14] assessed systems biology approaches for reverse-engineering and modeling (see also [13]). To recover a regulatory interaction network, the authors used three well-established reverse-engineering approaches: ordinary differential equations (ODEs), Bayesian networks and information theory. A gene synthetic network in the yeast consisting of 5 genes with 8 known interactions was investigated. From the results, the authors found ODEs and Bayesian networks could correctly infer most regulatory interactions from the experimental data with best values of PPV = 0.75 [Positive Predictive Value] and Se = 0.5 [Sensitivity]. In order to validate our approach, we applied conditional Granger causality [12] to the same experimental data. From our results, we found that the conditional Granger causality approach could also correctly infer most regulatory interactions and outperformed all the other three approaches reported in [14] with the best values of PPV = 0.83 and Se = 0.83. Hence the Granger causality approach, although simple, can be successfully applied to recover the network structure from temporal data and it could play a significant role in systems biology [21].

Initially, we applied conditional Granger causality to the switch-off time series which contained more time points than switch-on time series. The switch-off experiment data consisted of 4 replicates. Since a shift from galactose-raffinose- to glucose-containing medium caused a large initial decay, we simply removed the first two time points for 2 replicates. The time series were not stationary. The gene expression level decreased with time because of the inhibition effect of galactose-raffinose-containing medium. In order to apply conditional Granger causality, we were required to use ARIMA (Auto-Regressive Integrated Moving Average) rather than ARMA model to fit the data. The level of difference for ARIMA was chosen to be 1.

**Figure 3:**
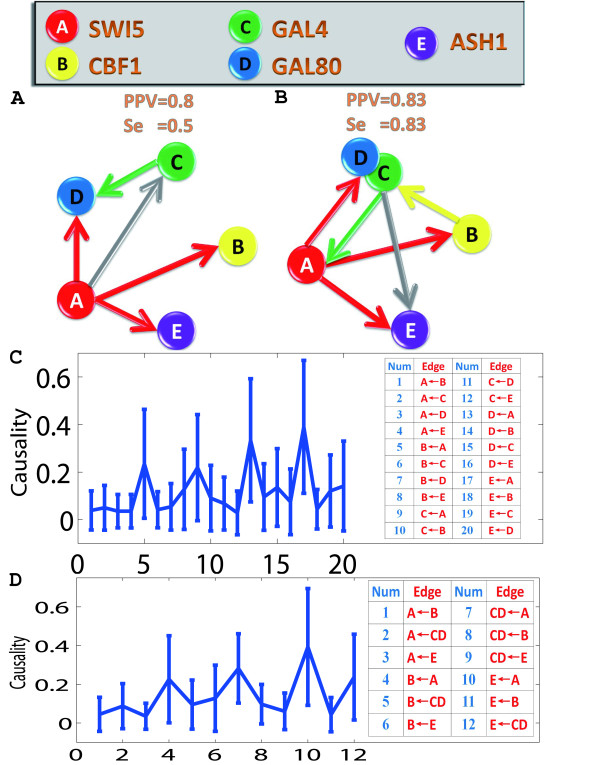
**Conditional Granger causality approach applied on experimental gene data**. The experiment measured the expression level of 5 genes after a shift from galactose-raffinose- to glucose-containing medium. The regulatory network was inferred by using conditional Granger causality approach. Solid gray lines represent inferred interactions that are not present in the real network, or that have the wrong direction (FP false positive). PPV [Positive Predictive Value = TP/(TP+FP)] and Se [Sensitivity = TP/(TP+FN)] values show the performance of the algorithm for an unsigned directed graph. TP, true positive; FN, false negative. (**A**) The network structure of 5 genes derived by conditional Granger causality. (**B**) Gal4 and Gal80 were grouped as a single node, so that only transcriptional regulation interactions are represented. (**C**) Conditional Granger causality results for 5 genes. The 95% confidence intervals graph, which is constructed by using bootstrapping method, is plotted. (**D**) Conditional Granger causality results for a grouped genes (Gal4 and Gal80 are grouped). The 95% confidence intervals graph, which is constructed by using bootstrapping method, is plotted.

Firstly, we used the conditional Granger causality approach to infer regulatory interactions for 5 genes. By using the bootstrapping method, we constructed 95% confidence intervals as shown in Fig. 3C. From this figure, we then constructed the causal network, which is displayed in Fig. 3A. Only the 5 most significant edges are shown in this graph. From this causal network, there are 4 true-positive edges and 1 false-positive edge. Our approach performs better: the PPV is 0.8, instead of 0.6 and the Se is 0.5, instead of 0.38.

We then grouped Gal4 and Gal80 as a single node as they form a complex [14], and then applied conditional Granger causality approach. Fig. 3D. shows 95% confidence intervals for the causality. From this figure, we can then recover a simplified causal network as shown in Fig. 3B. It shows the 6 most significant edges. There are 4 true-positive edges and 1 false-positive edge. By comparing our PPV (0.83) and Se (0.83) values with the original paper (PPV = 0.75, Se = 0.5), it is further confirmed that the performance of our algorithm is much better. The reason why the Granger causality outperforms the other approaches is clear from the detailed analysis in [12] where we have reported that the Granger causality is better than the Bayesian approach provided the data set is long enough. The Bayesian approach is similar to the ODE approach as claimed in [12]. Hence we could reasonably expect that the Granger approach is the best among the four approaches.

### Local Network: A Local Circuit of Seven Proteins

**Figure 4:**
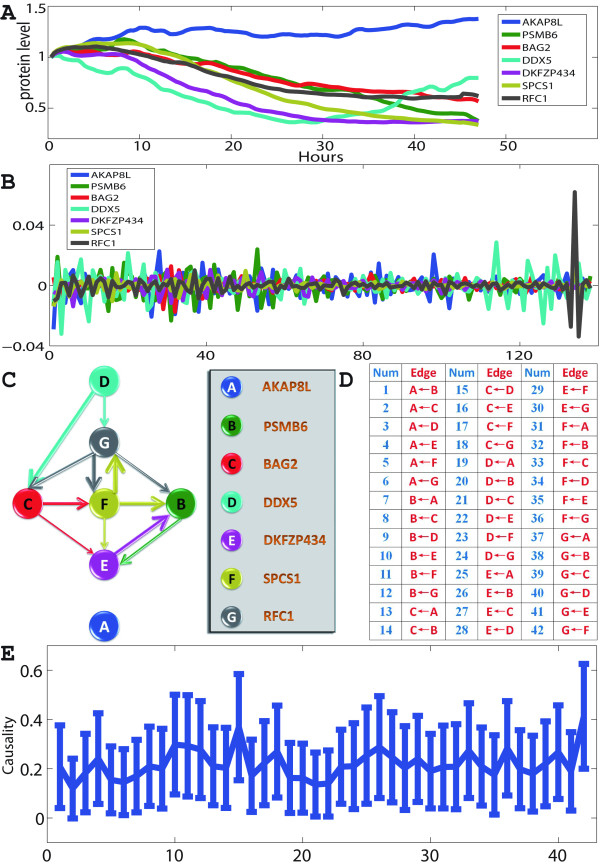
**Conditional Granger causality approach applied on experimental protein data**. The experiment measured the levels of 7 endogenously tagged proteins in individual living cells in response to a drug. (**A**) The time traces of 7 proteins are plotted. There are 141 time points. The time interval is 20 minutes. (**B**) ARIMA model is used to fit the data. We applied term-by-term differencing 3 times to the data. (**C**) The network structure for 7 proteins derived by using conditional Granger causality approach. (**D**) For visualization purpose, all directed edges (causalities) are sorted and enumerated into the table. (**E**) Conditional Granger causality results. The 95% confidence intervals graph, which is constructed by using method bootstrap, is plotted.

After testing our approach in the gene circuit, we applied conditional Granger causality approach on dynamic proteomics of individual cancer cells in response to a drug treatment [15,22]. In the experiment, an anticancer drug, camptothecin (CPT), with a well-characterized target and mechanism of action was used to affect the cell state. The drug is a topoisomerase-1 (TOP1) poison with no other target, which can eventually cause cell death. To follow the response to the drug, 812 different proteins in individual living cells were measured with a time interval of 20 minutes. A total number of 141 sample points (more than 40 hours) were collected. This dataset, much larger than the gene data reported above, gives us the opportunity to construct both local and global networks. In [15], seven proteins were investigated in more details, including two proteins (DDX5 and RFC1) that were reported to be essential. Fig. 4A shows the time traces of the seven proteins, denoted as X. They clearly are not stationary, a property that is required for Granger Causality. To overcome this, the model used to fit the time series is changed from ARMA (Autoregressive moving average model) to ARIMA (Autoregressive integrated moving average). Crucially, this transformation does not impact on the true connections between elements. Fig. 4B shows the transformed data, obtained after differencing the original data term by term 3 times. Fig. 4E. shows the Granger causality found for all possible pairs of proteins, together with their 95% confidence intervals calculated though a bootstrap. From the figure, we can then construct the causal protein-interaction network, which is displayed in Fig. 4C. Only the 12 most significant edges are shown in this graph. In the literature, it has been reported that the protein DDX5 was significantly correlated with the cell fate (with a p-value *p *< 10^-13^). It has been further proved that it plays a functional role in the response to the drug: a doubling in the death rate was observed during the first 40 hours when DDX5 was knocked-down [15]. Protein RFC1 also showed a significant correlation with cell fate (with a p-value *p *< 10^-6^). Our derived network is in good agreement with these two biological characteristics. Protein DDX5, which is the most significantly correlated with the cell fate, is on the top level of the network. Protein RFC1 is in a lower level comparing to DDX5, since the causal relation is from DDX5 to RFC1. Therefore, the results on the proteomic data and gene data confirm that the Granger causality is a simple and accurate algorithm to recover the network structure.

**Figure 5:**
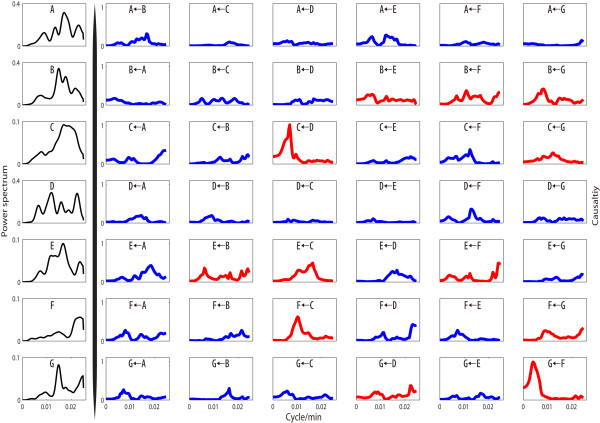
**Conditional Granger causality in frequency domain**. Conditional Granger causality was applied to experimental data in the frequency domain and power spectrum density analysis for 7 proteins (the most left column in black line). The significant causalities are shown in red lines in the figure.

Fig. 5. shows the same analysis in the frequency domain. From the result, we find that there are strong interactions from D (DDX5) to C (BAG2) at around 0.006 cycle/min or one cycle every three hours. From the power spectrum result for D and C, we can also find an energy peak at this frequency. In addition, there is a strong chain interaction from D to G (RFC1) via C and F (SPCS1). This chain contains the 3 strongest interactions. Each element in the chain affects its downstream element at a similar frequency.

### Global Network: Synthesized Data

To measure the performance of the Global Granger Causality algorithm introduced in this paper, we first consider some toy models. The first toy model is a high-dimensional time series. We also compare the result of GGC with that of PGC.

#### Example 1

**Figure 6:**
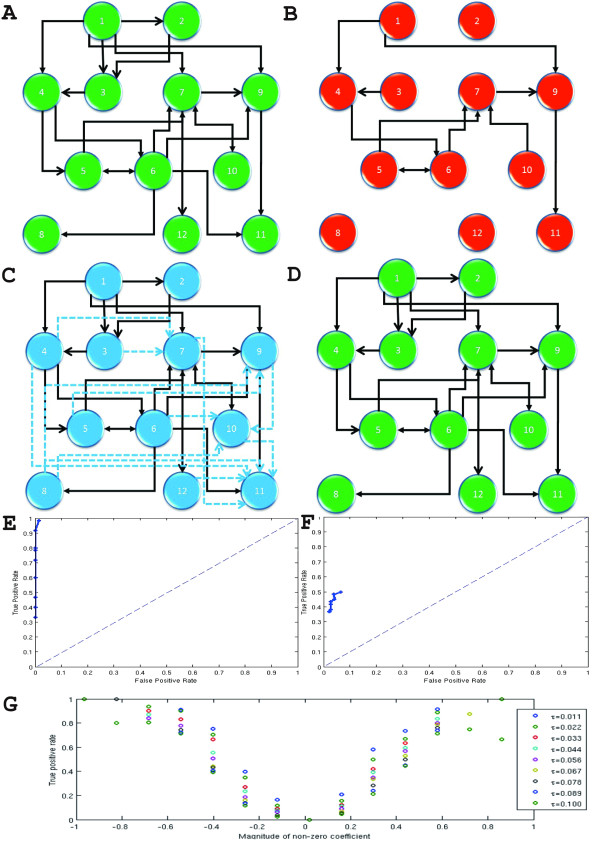
**Global Granger Causality (GGC) algorithm applied on a simple toy model**. (**A**) The actual network structure used in toy model of global network. (**B**) Network structure inferred from PGC. (**C**) Network structure inferred from pair-wise Granger causality (solid and dashed links). By using partial Granger causality among three units, we can delete some of them (dashed links). (**D**) The final network structure from GGC, it is consistent with the actual relationship. (**E**) ROC curve summarizing the performance of the procedure on a random network with maximum non-zero coefficients. (**F**) ROC curve summarizing the performance of the algorithm on a random network with random non-zero coefficients. (**G**) True positive rate as a function of the magnitude of the non-zero coefficient.

Suppose that 12 simultaneously generated time series were generated by the equations:(16)

where *ω*_1_,⋯,*ω*_12 _are zero-mean uncorrelated process with identical variance. We generated time series of 80 points. The true network structure is depicted in Fig. 6A, there are 21 actual links. We first applied PGC to the data directly and used a bootstrap method to construct the network structure. More specifically, we simulated the fitted VAR model to generate a dataset of 1000 realizations of 80 time point, and used 3*σ *as the confidence interval. If the lower limit of the confidence interval was greater than zero, we considered there was a relationship between two units. The network structure is depicted in Fig. 6B. The network structure we obtained from PGC was misleading. The reason is that since the order of the model is 4, the number of total parameters we should estimate in this model is 12 × 12 × 4, the estimator is unreliable with such little data.

Secondly, we used GGC to investigate the network structure. Fig. 6C shows the results we obtained after applying pairwise Granger causality. There are 33 links in total. We computed partial Granger causality conditioned on any intermediate node to identify whether the links appearing in Fig. 6C are direct or indirect. If the lower limit of the confidence interval of partial Granger causality is less than zero, then the link is regarded to be indirect and is deleted from Fig. 6C (dashed arrows). Fig. 6D is the final structure we get from GGC; it is consistent with the actual structure Fig. 6A.

#### Example 2: Random network

Next we present a validation of our method with a series of experiments on random networks for which the true structure is known. We built an Erdös-Rényi random graph with N = 200 nodes and M = N × log(N) = 1060 edges. From the network structure, we generated N time series with an auto-regressive model of order 1 whose transition matrix is the transpose of the adjacency matrix of the network, with its largest eigenvalue normalized to 0.99 to obtain a stable system. Each time series was 200 time-points long and normal noise of unit variance was added throughout. The algorithm was applied to each single node to get a list of their guessed ancestors. We then compared the true and computed lists of ancestors. One should expect that the connection between two nodes to be difficult to uncover if the corresponding coefficient in the linear model is small. To factor this out, we first considered the case where the non-zero coefficients of the transition matrix were all equal and maximized. We then applied the method on the case where the non-zero coefficients were randomly distributed.

#### Example 3: Constant coefficients

The data were generated by an auto-regressive model with transition matrix A. A is a scaled version of the transpose of the true adjacency matrix. The scaling factor was chosen so as to be maximal while leading to a largest eigenvalue for A of less than 1 (or the model degenerates). In this particular case, it was found to be 0.1736. The procedure has one parameter τ, the threshold at which a Granger-causality is deemed significant. By varying this parameter from 0 to 0.1, we obtained different large networks which we compared to the truth. The accuracy of each network was summarized by its true positive and false positive rates. Fig. 6E shows the resulting receiver operating characteristic (ROC) curve that is the graph obtained by plotting the false positive rate against the true positive rate. The performance of the method was extremely good, with an area under the curve close to 1. Crucially for biological applications, the false positive rate is always very small.

#### Example 4: Random coefficients

In this setup, the non-zero coefficients of the transition matrix were randomly distributed (normally distributed with mean 3 and multiplied by -1 with probability 1/2). The matrix was then scaled in the same manner as before. Fig. 6F shows the performance of the method on this harder problem. The method is not as accurate as before, with a maximum true positive rate just over 0.5. However, the false positive rate is still very low: the method doesn't guess as many ancestors as before but its guesses are rarely wrong. The fact that more connections are now missed out is not surprising: the non-zeros coefficients are randomly distributed and can be very small. Fig. 6G shows how the true positive rate varies with the magnitude of the coefficients; the true positive rate goes to zero with small coefficients.

### Global Network: A Global Circuit of 812 Proteins

**Figure 7:**
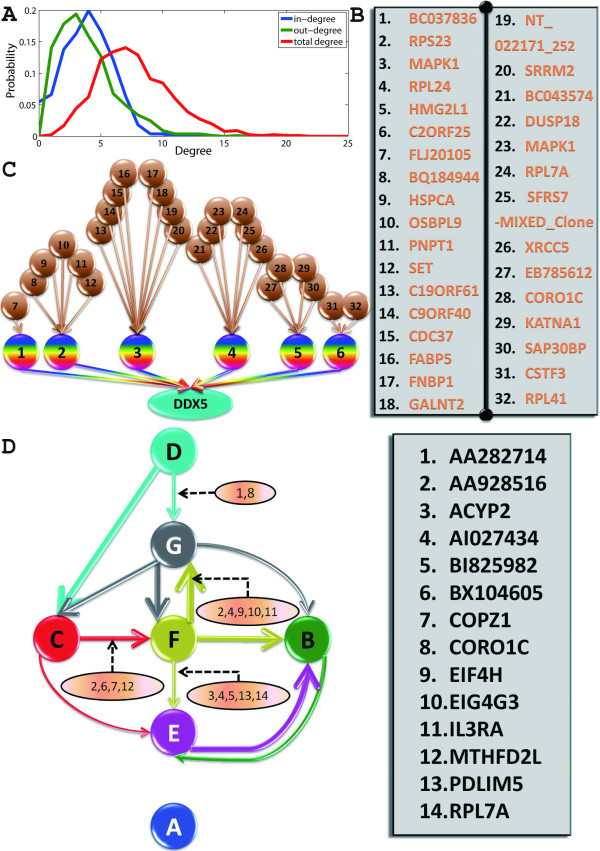
**Global Granger Causality algorithm applied on experimental data for global network re-construction**. (**A**) In-, out- and total degree distributions of the large network calculated from the whole dataset. (**B**) For visualization purpose, the proteins are enumerated into the table (**C**) Direct ancestors of the protein DDX5: BC037836, C2ORF25, HMG2L1, MAPK1, RPL24 and RPS23.(**D**) External influences identified by the second iterative procedure, in brown ovals.

**Figure 8:**
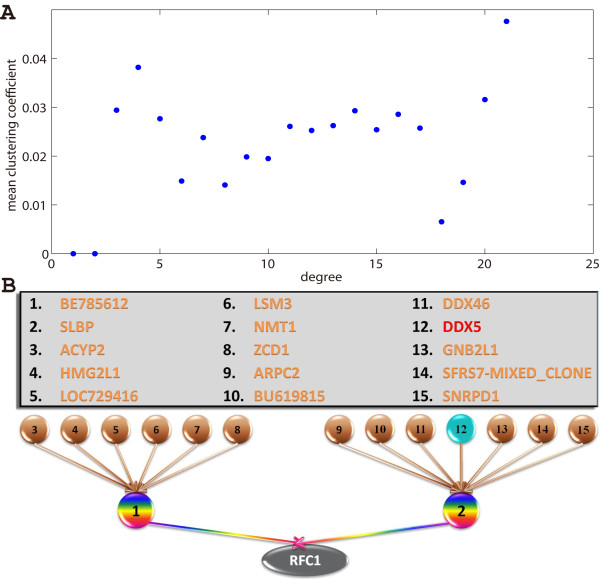
**Global Granger Causality algorithm applied on experimental data for global network re-construction**. (**A**) The overall mean clustering coefficient (the probability of neighbours being inter-connected) is an order of magnitude larger than the one of a random network (0.022 instead of 1/768 = 0.0013). But the network is not modular: the mean clustering coefficient with respect to degree is more or less constant. (**B**) Direct ancestors of RFC1, as well as their own direct ancestors. The causal link from DDX5 to RFC1 is now completely identified: an intermediate protein (SLBP) connects them.

We then applied our GGC approach on the whole dataset of 812 proteins on dynamic proteomics of individual cancer cells in response to a drug treatment. Fig. 7C shows the direct ancestors of protein DDX5, known to be at the top level of the circuit, as shown in the previous section. Our result suggests that controlling for either BC037836, C2ORF25, HMG2L1, MAPK1, RPL24 or RPS23 will have an effect on DDX5 and thus on the whole circuit. A similar figure for RFC1 is shown in the Fig. 8.

Setting the same threshold as the one used to obtain the small circuit, a large, sparse network is obtained: 768 nodes remain (discarding those with no connections) and 2972 edges were found, which represented only 0.5% of all the possible edges. The complete structure can be found in the **Additional File **1. Fig. 7A shows the distributions of in-, out- and total degree of the nodes. All three distributions are exponential, precluding the possibility of a scale-free network. Each node has an average in-degree and out-degree of 3.8, indicating a well-connected network. This is confirmed by the characteristic path length (average of the shortest path between all pairs of nodes). Considered undirected, the graph has a characteristic path length of 3.8, in line with those of previously reported biological networks (see [23] and references within), including protein-protein interaction networks, although it should be noted that the present study is concerned with the dynamics of the proteins (as in [24] for example) and not their physical interactions (in which case the network is undirected by construction). The directed graph also has a small characteristic path length of 5.7 nodes and a small diameter (largest shortest path) of 12 nodes. Such connectedness indicates that the network is a small world [25,26]. However, it is not particularly modular: while its mean clustering coefficient is an order of magnitude (17 times) higher than one of a random network, the clustering coefficient is almost constant with respect to the node's degree. In other words, the same level of clustering is found everywhere regardless of the node's degree.

The previous small network in Fig. 4 was obtained by using the conditional granger causality. Conditional Granger causality can be misled by common influence: if both nodes are subjected to an unknown common source, it can have an effect on their connections. Partial Granger causality - another extension of Granger causality [16,27,28] - can address this issue by considering an unseen external input in the linear model and working out its effect on the connection. For example, the partial Granger causality between DDX5 and RFC1 is very small, even though the conditional Granger causality between them is high (Fig. 4) and there exists a short path (1 intermediate) from DDX5 to RFC1 in the large network. This suggests the connection is affected by a common unseen source.

In order to identify which proteins have an influence on the connections between the 7 proteins of interest (AKAP8L, PSMB6, BAG2, DDX5, DKFZP434, SPCS1, and RFC1), we first extracted them as well as the proteins belonging to the shortest paths between them, resulting in a subset of 118 proteins. We then applied a filtering process on each of the 12 connections uncovered in the previous section. The rationale of the algorithm is that if removing the (explicit) influence of a protein makes the connection between two nodes change, then this protein should be kept as a potential influence on the connections - if z is independent of x and y, then z does not affect the Granger causality and F_x→y|z _= F_x→y_. After filtering for those that have an influence, we then considered their pairs and build a new subset, then triplets etc.. The end-result is a set of proteins which have a substantial influence on the connection.

Fig. 7D shows the small network of 7 proteins with the now-identified external influences. Note that those proteins do not necessarily belong to the path from one node to the other, but rather they have some substantial influence on the connection as a whole, for example on some members of the path.

### How reliable is Global Granger Causality?

In theory, we can recover all possible links from the pairwise Granger causality procedure and have to Granger-condition on all combinations of the nodes in the system to remove an indirect connection. However, it is an NP-hard problem and we will stop at a stage k, i.e., we only need to Granger-condition on the combinations of up to k nodes. Therefore, the analysis on how to choose k and the probability of correctly uncovering the true relationship of the whole network when we stop at stage k is of vital importance. In this section, we will provide some simulation and theoretic results on these questions.

Consider a network with N nodes {X_1_,⋯,X_N_} with a connection probability p. There are N × (N - 1) × p direct links on average in the whole system. We intend to estimate how many indirect connections are left when we stop at stage k. Here we focus on a pair X to T, where X, T are in {X_1_,⋯,X_N_}. If there exists only one single path from X to T, this link can be discarded by Granger-conditioning on a single intermediate node in the path. If there are more than one paths from X to T, in theory, this link should be discarded by Granger-conditioning on all the other nodes.

***Definition 1 (bottleneck)**. Assume that there are m distinctive directed paths from S *∈ {X_1_,⋯,X_*n*_} *to T and p(S, T) be the set of all distinctive directed paths from S to T. A set of nodes *{Z_1_,⋯,Z_m_} *is called a section from S to T if there is no directed path from S to T in the graph *{X_1_,⋯,X_N_}-{Z_1_,⋯,Z_m_}. *A section which minimizes its total number of elements of the section is called a bottleneck*.

For example, in Fig. 1C both {B_1_, B_2_} and {B_3_} are sections from S to T, but {B_3_} is the bottleneck..

***Lemma 1**. Assume that the set *{B_1_,⋯,B_m_} *is the bottleneck from S to T, we have*

**Proof**. We only check two cases here. The first case is that there is a single serial connection from S to T. For example, we have *S *→ *B*_1 _→ *B*_2 _→⋯*B*_*n *_→ *T *where every single node {B_i_} is a bottleneck of the path. If we condition on one of the single node B_i _in the path, we need to show

According to the definition, we need to find the autoregression expression:

where Γ is the delay operator and C, D are polynomials, *ξ *is the noise term. From the assumption of the path structure, we conclude(17)

Therefore(18)

where E, F, G are polynomials and *ε *is the noise (could be different). From the equation above, we see that for any node B_i, _we have . Intuitively, in a serial path *S *→ *B*_1 _→ *B*_2 _→⋯*B*_*n *_→ *T*, the information cannot be transmitted from S to T if B_i _is removed. In conclusion, for a single path, the Granger causality is zero whenever we condition on one of its nodes in the path. It is not necessary to condition on the whole path to remove the causality.

The second case is as depicted in Fig. 1C. There are two different paths from S to T, B_1 _and B_2 _converge to a common bottleneck B_3_. It is easy to see that information can not be transmitted from S to T if B_3 _is removed, then we can easily see that

Combining the above two cases completes the proof of the lemma.

Lemma 1 tells us that if there are m distinctive paths from S to T, i.e., the number of the bottleneck is m, then the causality between S and T will vanish when we take into account the partial Granger causality on {X_1_, ...,X_m_}. There may exist other common drives to the observed nodes S and T such as Fig. 1D. We assume the partial Granger causality can delete the influence of such drive and exclude such case in our analysis.

The exact formula of the number of bottlenecks seems to be fairly complicated but we can have a first look at the empirical distribution of it. For a variety of connection probability p, we generate 500 random networks when N = 100. For each network, we randomly select two nodes and compute the number of the bottleneck between them. Fig. 1E shows the histograms when p = 0.015, 0.02, 0.03 and 0.05, respectively. From these figures, it can be easily seen that the sparser the network is, the quicker we can detect the true structure from global Granger causality. When p = 0.015, it is very likely for any two nodes to be unconnected or directly connected, then almost all the true relationships can be uncovered at stage 1. When p = 0.02, all the true relationships can be uncovered at stage 2. When p = 0.03, the probability of uncovering the true relationship is 90.8% at stage 2 and 98.6% at stage 3. When p = 0.05, the probability of uncovering the true relationship is 82.2% at stage 4 and 97.8% at stage 6. It is not until stage 9 that all indirect links can be discarded.

## Conclusion

In this paper, we focused on the Granger causality approach in both the time and frequency domains in local and global networks. For a local gene circuit, a recent *Cell *paper by Irene Cantone *et al*. [14] assessed systems biology approaches for reverse-engineering and modeling by investigating a gene synthetic network in the yeast consisting of 5 genes with 8 interactions (also see highlight [13]). From our results, we found that our conditional Granger approach could also correctly infer most regulatory interactions and outperform the three approaches reported in the [14]. For a local protein-interaction network, our derived network is in good agreement with biological characteristics. Therefore, the results on the proteomic data and gene data confirm that the Granger causality is a simple and accurate approach to recover the network structure. For a global network, our novel approach was successfully used to build a large network from all the recorded 812 proteins.

## Authors' contributions

The study of the local network in the time and frequency domain was carried out by CZ. The study of the global network was carried out by CL and SG. The whole article was supervised by JF. All authors read and approved the final manuscript.

## Supplementary Material

Additional file 1 **The global network derived by Global Granger causality algorithm**. The re-constructed global network is stored in PDF format. Click here for file
