# Supplementary figures and images for: Identifying interactions in the time and frequency domains in local and global networks - A Granger Causality Approach
Source: BMC Bioinformatics. 2010 Jun 21;11:337. doi: 10.1186/1471-2105-11-337 (PMC2897832; doi:10.1186/1471-2105-11-337)

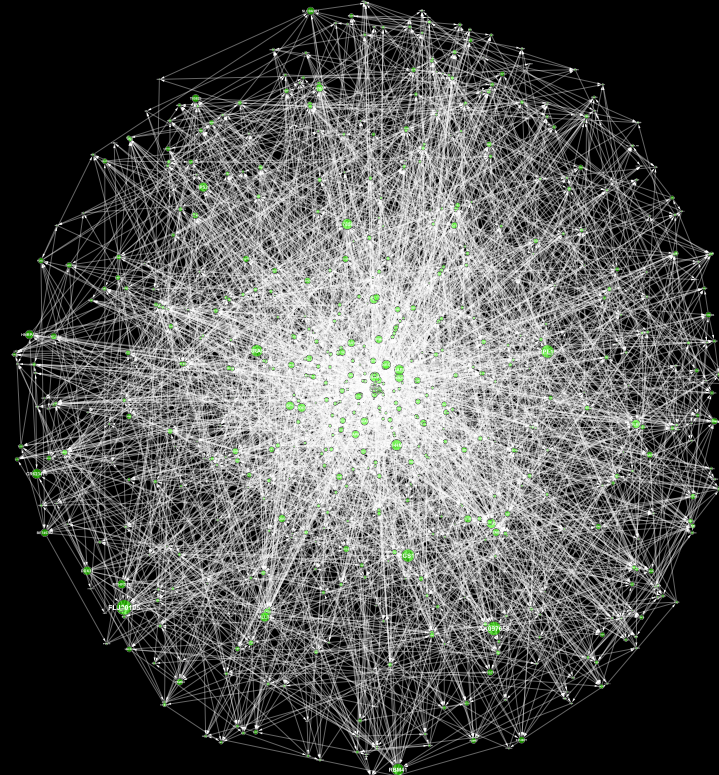

Supplement: Additional file 1 — The global network derived by Global Granger causality algorithm. The re-constructed global network is stored in PDF format. [file 1471-2105-11-337-S1.PDF]
